# The effects of lower eyelid epiblepharon surgery on the meibomian glands

**DOI:** 10.1186/s12886-025-03940-0

**Published:** 2025-03-07

**Authors:** Seongmi Kim, Da Eun Yoon, Hyun Sun Jeon, Namju Kim

**Affiliations:** 1 Department of Ophthalmology, Seoul National University College of Medicine, Seoul, Korea; 2 Department of Ophthalmology, Seoul National University Bundang Hospital, Seongnam, Korea; 3 Department of Ophthalmology, Jeju National University College of Medicine, Jeju-si, Korea; 4 Department of Ophthalmology, Jeju National University Hospital, Jeju-si, Korea; 5 Department of Ophthalmology, Seoul National University Bundang Hospital, 82, Gumi-ro, 173 Beon-gil, Bundang-gu, Seongnam, Gyeonggi-do 13620 Korea

**Keywords:** Meibomian gland, Epiblepharon, Meibography, Astigmatism

## Abstract

**Background:**

To investigate the morphological and functional changes of meibomian glands (MG) in pediatric patients who underwent surgery for lower eyelid epiblepharon.

**Methods:**

A total of 176 eyes of 88 patients aged 19 and under (mean age: 8.9 ± 2.8 years old) who underwent bilateral lower eyelid epiblepharon correction surgery from May 2022 to April 2023 were included. Meibograde, lipid layer thickness (LLT), total blink rate, and corneal/refractive astigmatism were compared between pre- and 2 months postoperatively.

**Results:**

There were no statistically significant changes in meibograde and LLT after surgery. The total blink rate was significantly decreased after surgery (*p* = 0.02). While corneal and refractive astigmatism showed no significant changes in total eyes, corneal astigmatism in eyes of high astigmatism of 2.0D or more subgroup exhibited a significant decrease postoperatively (*p* < 0.001).

**Conclusions:**

Lower eyelid epiblepharon surgery in pediatric patients does not significantly alter the structure and function of the MG. Additionally, stabilizing the ocular surface through surgical correction may have beneficial effects on blink rate and corneal astigmatism. Although the long-term evaluation would be needed, we suggest that lower eyelid epiblepharon surgery could be performed without worrying about adverse effects on the MG.

## Background

Epiblepharon is a common eyelid condition characterized by horizontal skin folds that may extend over the eyelid margin, causing the eyelashes to touch the corneal surface [1]. This condition is frequently observed in both lower eyelids of East Asian children and is traditionally managed through surgical intervention [2, 3]. The corrective surgical procedure for lower eyelid epiblepharon involves suturing the subcutaneous tissue of the upper skin edge and the lower margin of the tarsus to achieve outward rotation of the eyelashes [4–6]. However, concerns have been raised regarding the potential impact of this surgical procedure on meibomian glands (MG) located within the tarsus.

The MG, which is located within the tarsal plate of both the upper and lower eyelids, plays a crucial role in maintaining tear film stability and overall ocular surface health. A previous study by Christoph et al. [7] demonstrated an improvement in MG function after the implementation of a lateral canthal sling procedure in patients with age-related lid laxity. Conversely, Vaidya et al. [8] examined changes in MG function following eyelid corrective surgery in patients with involutional entropion and concluded that there were no significant postoperative alterations in MG function. However, there is a significant gap in our understanding of how morphology or function of MG are affected after surgical correction of the lower eyelid epiblepharon, as no study has specifically addressed this issue. Therefore, the primary objective of this study is to assess and compare morphological and functional changes of MG in pediatric patients who underwent surgery for lower eyelid epiblepharon, using the LipiView^®^II ocular surface interferometer.

## Methods

### Population

This retrospective study was approved by the Institutional Review Board of the Seoul National University Bundang Hospital (IRB No. B-2401-875-106), and adhered to the tenets of the Declaration of Helsinki. The study included patients aged 19 and under who underwent lower eyelid epiblepharon surgery at Seoul National University Bundang Hospital from May 2022 to April 2023.

Surgical indications for congenital lower eyelid epiblepharon were significant irritation symptoms and corneal erosions matching the extent of the epiblepharon. All patients underwent surgical correction of bilateral lower eyelid epiblepharon. Informed consent for surgical procedures was obtained from the parents of all patients. All surgeries were performed by a single experienced oculoplastic surgeon (NK) using the rotating suture technique.

**Fig. 1:**
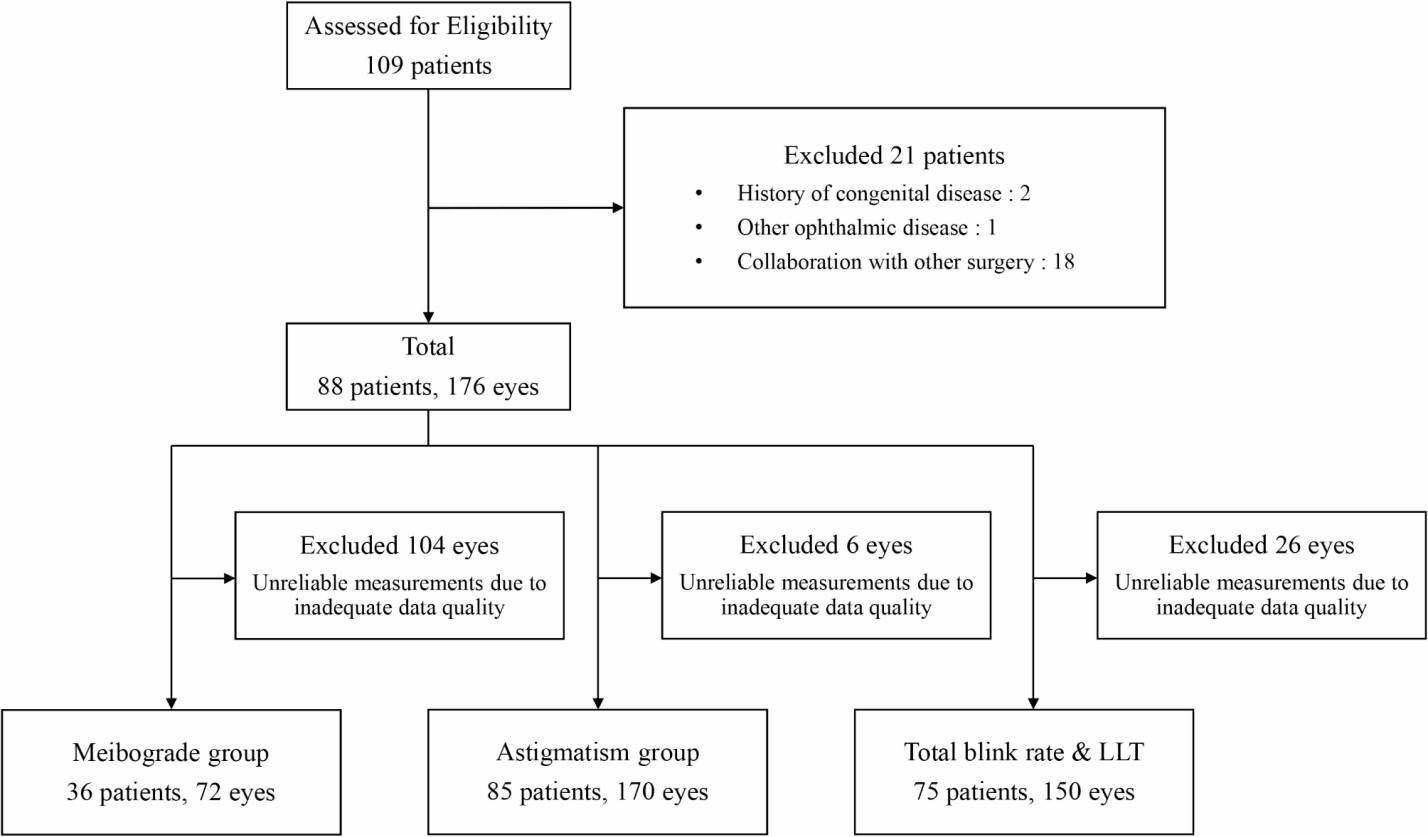
Flow diagram illustrating patient selection and allocation for postoperative assessments. Among a total of 109 patients who underwent lower eyelid epiblepharon surgery who were eligible for the study, 21 were excluded based on the following exclusion criteria: two had congenital diseases, one had a concurrent ophthalmic disorder, and 18 had collaboration with strabismus surgery. Finally, 176 eyes of 88 patients who met the inclusion criteria were included. Among them, meibography was available for 72 eyes of 36 patients, topography for 170 eyes of 85 patients and total blink rate and lipid layer thickness for 150 eyes of 75 patients, excluding inadequate data quality for each, respectively

Patients with a history of congenital diseases, those concurrently afflicted with other ophthalmic conditions, and those undergoing collaborative surgery involving additional ophthalmic procedures were excluded from the study. Additionally, eyes with unreliable measurements due to inadequate data quality in both preoperative and postoperative examinations as well as cases lost to follow-up were excluded from the analyses (Fig. 1).

### Surgical technique

**Fig. 2:**
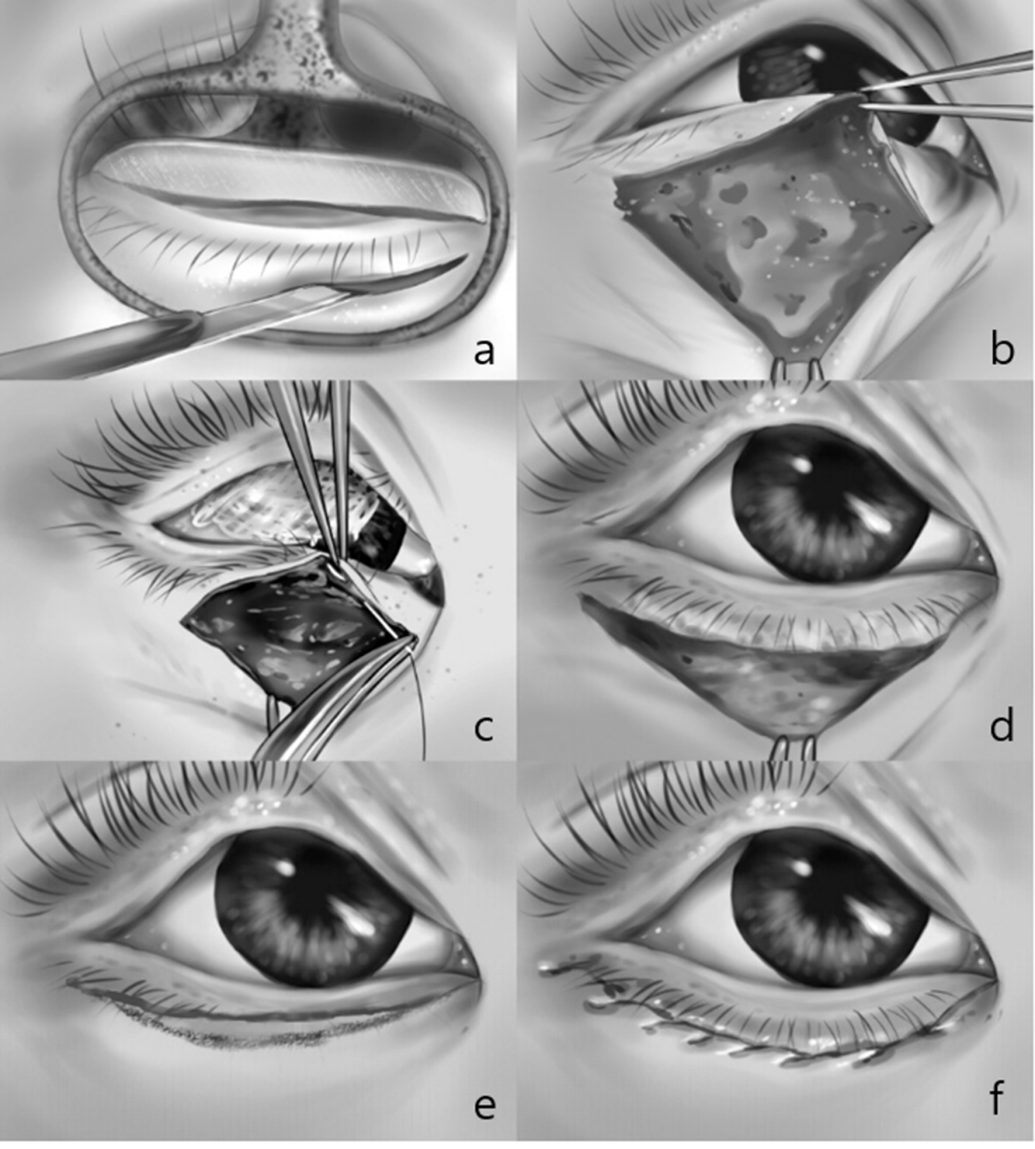
Surgical procedure. The subciliary skin was incised with a No.15 scalpel blade, while the lid was held using chalazion forceps (**a**). Dissection was performed inferiorly between the orbicularis muscle and tarsus until the lower margin of the tarsus was exposed (**b**). The subcutaneous tissue at the upper edge of the incised subciliary skin was fixed to the lower margin of the tarsus (**c**). The eyelid was directed outward by securing it with nylon interrupted sutures on the tarsus (**d**). The lower edge of the incised skin was lifted to overlap the upper skin edge, and redundant skin was excised (**e**). The skin was closed continuously (**f**)

All surgeries were performed under general anesthesia. A subciliary skin incision line was marked horizontally from the temporal point of the inferior punctum along the entire lid length, 1 mm below the ciliary line. Local infiltration of 2% lidocaine mixed with epinephrine at a ratio of 1:100,000 was given subcutaneously along the marked line. Following skin preparation, the upper eyelid was retracted superiorly to avoid obscuring the margin of the lower lid by the cilia of the upper lid. The subciliary skin was incised along the previous mark with a No.15 scalpel blade, while the lid was held using chalazion forceps. The chalazion forceps were removed and dissection was performed inferiorly between the orbicularis muscle and tarsus using monopolar cautery until the lower margin of the tarsus was exposed. The pretarsal orbicularis oculi muscle remaining beneath the upper edge of the skin incision was excised using Westcott scissors until the tarsal plate was more exposed. The subcutaneous tissue at the upper edge of the incised subciliary skin was fixed to the lower margin of the tarsus using five to seven interrupted 8–0 nylon sutures to ensure outward eversion of the cilia. The lower edge of the incised skin was lifted to overlap the upper skin edge. A line was drawn on the overlapping lower skin to match the upper skin wound edge under it, and the redundant tissue was excised using Stevens’ scissors. The skin was closed continuously with 5–0 rapid vicryl in young patients and with 6–0 black silk in patients older than 10 years of age. At the conclusion of the procedure, a small amount of antibiotic ointment was applied to the skin wound, and cold compressions were applied for the first 12 h postoperatively (Fig. 2).

### Data collection

Patient demographic information, including age and sex, was also obtained. Patient data were collected before surgery and at 2 months postoperatively. The outcome measures included meibography, lipid layer thickness (LLT), total blink rate, corneal astigmatism, and refractive astigmatism. Meibography, LLT, and total blink rate were measured using a LipiView^®^II ocular surface interferometer (TearScience Inc, Morrisville, NC, USA). Corneal astigmatism was measured using an OPD-Scan III (Nidek Technologies, Gamagori, Japan) and refractive astigmatism was measured using a KR-800 A (Topcon, Tokyo, Japan).

### The ocular surface interferomter procedure

The patient undergoes measurements of lipid layer thickness, blinking, and Meibography using the LipiView^®^II. Initially, the patient fixates on a target light while reflective images of the eye are captured. Subsequently, the patient is instructed to blink naturally for a duration of 20 s, during which video is recorded to assess lipid layer thickness and blinking patterns.

To obtain meibography of the lower eyelid, the lid everter was placed on the lower eyelid, and the eyelid was pulled down and everted with the instrument in contact, ensuring the full extent of the meibomian glands was visible. For upper eyelid meibography, the upper eyelid was everted using a cotton tip or fingers.

### Grading of meibomian gland dropout

As we included patients who underwent epiblepharon surgery on both lower eyelids, the assessment of the meibograde was concentrated exclusively on the lower lid. The grading system for partial or complete loss of the MG in the lower eyelid included the following four categories: grade 0 (no loss of the MG); grade 1 (area loss comprising less than one-third of the total MG area); grade 2 (area loss ranging from one-third to two-thirds); and grade 3 (area loss exceeding two-thirds) [9].

### Statistical analysis

IBM SPSS version 27.0 (IBM Corp., Armonk, NY, USA) was used for the statistical analyses. A paired t-test was performed to compare the preoperative and postoperative values, and quantitative variables were presented as mean and standard deviation (SD). A *p*-value of less than 0.05 was considered statistically significant.

## Results

Of the 109 patients who underwent epiblepharon surgery, 21 were excluded based on the following exclusion criteria: two had congenital diseases, one had a concurrent ophthalmic disorder, and 18 had collaboration with strabismus surgery. Finally, 176 eyes of 88 patients (mean age: 8.9 ± 2.8 years old, range 3 to 18 years) who met the inclusion criteria were included. Among them, meibography was available for 72 eyes of 36 patients (40.9%), total blink rate and LLT for 150 eyes of 75 patients (85.2%), and topography for 170 eyes of 85 patients (96.6%).

There was no statistically significant difference between preoperative meibograde (0.31 ± 0.64) and postoperative meibograde (0.33 ± 0.65; *p* = 0.42, Table 1).

**Table 1:** Change of clinical parameters after lower eyelid epiblepharon correction surgery

Variable		*N* (eyes)	Preoperative	Postoperative	*p*-value
Meibograde		72	0.31 ± 0.64	0.33 ± 0.65	0.42
Lipid layer thickness (nm)		150	66.30 ± 27.36	66.59 ± 24.83	0.91
Total blink rate		150	8.03 ± 5.04	6.85 ± 4.91	0.02^*^
Astigmatism (D)	Corneal Astigmatism	170	1.95 ± 1.63	1.77 ± 1.47	0.20
	Refractive Astigmatism	170	1.04 ± 1.04	1.05 ± 1.09	0.75
High Astigmatism (D) (Corneal Astigmatism ≥ 2.0D)	Corneal Astigmatism	57	3.64 ± 1.77	2.41 ± 1.03	< 0.001^*^
	Refractive Astigmatism	57	1.75 ± 1.36	1.83 ± 1.39	0.39
Low Astigmatism (D) (Corneal Astigmatism < 2.0D)	Corneal Astigmatism	113	1.10 ± 0.49	1.44 ± 1.55	0.02^*^
	Refractive Astigmatism	113	0.68 ± 0.56	0.66 ± 0.61	0.63

Values are presented as mean ± standard deviation

^*^Statistically significant differences between two groups (*p* < 0.05) by paired *t*-test

The LLT was not significantly different between preoperative (66.30 ± 27.36 nm) and postoperative measurements (66.59 ± 24.83, *p* = 0.91). However, the total blink rate was significantly decreased postoperatively (6.85 ± 4.91) compared to preoperatively (8.03 ± 5.04; *p* = 0.02, Table 1).

**Fig. 3:**
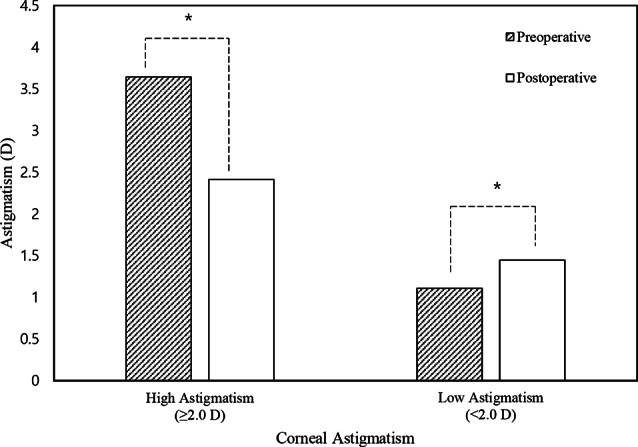
Mean changes in subgroups **A** and **B** between preoperative and postoperative groups. High Astigmatism is defined as ≥ 2.0 D, and Low Astigmatism as < 2.0 D. In the “high astigmatism group,” the corneal astigmatism was significantly decreased after surgery from 3.64 ± 1.77D to 2.41 ± 1.03D (*p* < 0.001). On the other hand, in the “low astigmatism group,” the corneal astigmatism was significantly increased after surgery from 1.10 ± 0.49D to 1.44 ± 1.55D (*p* = 0.02). *Statistically significant differences between the two groups were identified (*p* < 0.05) using a paired t-test

In all eyes, there were no significant changes in corneal or refractive astigmatism after surgery. Mean corneal astigmatism was 1.95 ± 1.63 diopter (D) and 1.77 ± 1.47 D before and after surgery, respectively (*p* = 0.20). Mean refractive astigmatism was 1.04 ± 1.04 D and 1.05 ± 1.09 D before and after surgery, respectively (*p* = 0.75, Table 1). We conducted a subgroup analysis by categorizing eyes into two subgroups based on the level of corneal astigmatism: a “high astigmatism group (57 eyes from 57 patients)” with corneal astigmatism equal to or exceeding 2.0D and a “low astigmatism group (113 eyes from 67 patients)” with corneal astigmatism below 2.0D. In the “high astigmatism group,” the corneal astigmatism was significantly decreased after surgery from 3.64 ± 1.77D to 2.41 ± 1.03D (*p* < 0.001, Fig. 3). However, in the “low astigmatism group,” the corneal astigmatism was significantly increased after surgery from 1.10 ± 0.49D to 1.44 ± 1.55D (*p* = 0.02, Fig. 3). Regarding refractive astigmatism, there was no statistically significant difference observed in both the “high” and the “low” astigmatism groups (*p* = 0.39 and *p* = 0.63, respectively) (Table 1).

## Discussion

During the epiblepharon surgery, there is a postulation that it may influence the structure of the MG. However, there is currently a lack of research in this regard. This is the first study to evaluate the morphological and functional changes in the MG after epiblepharon surgery. Our study confirmed that there were no significant changes in the meibograde or LLT after surgery, indicating that lower eyelid epiblepharon surgery does not have a substantial short-term impact on the structure or function of the MG.

In a healthy eyelid, the ductules of the MG contain keratohyalin granules, and their openings are located in front of the mucocutaneous junction. This positioning allowed for the direct delivery of meibum into the tear meniscus [10]. If epiblepharon surgery causes subtle structural changes in the MG, it could be hypothesized that it may potentially impact the function of the MG. In addition, the use of monopolar cautery during surgery may have had a thermal effect, albeit minimal, that could potentially affect surrounding structures, including the meibomian glands. Although we believe the effect to be negligible, we acknowledge this potential effect.

Meibography is a widely used diagnostic imaging technique for meibomian gland dysfunction (MGD). This enables the objective and reproducible observation of MG [11, 12]. While it is a short-term observation over a period of 2 months, our study is meaningful as the first report on the changes in MG by evaluating meibography following epiblepharon surgery, a procedure frequently performed in pediatric patients.

LLT is considered an indicator that plays a role in preventing tear evaporation in patients with dry eye, although the standard has not been clearly established [13]. Additionally, it is regarded as one of the indicators for assessing the function of the MG [14, 15]. Reduced LLT may be indicative of dry eye, and MGD is associated with their manifestations [14–17]. In a previous study, after the surgical correction of marginal entropion, repositioning of the MG ducts and orifices facilitated the release of pooled secretions, leading to an increase in LLT [18]. However, the results of this study indicated that there were no statistically significant changes in LLT after surgery. Considering that neither meibograde nor LLT showed postoperative changes, it can be inferred that, in the short term, surgery does not affect MG function.

The blink patterns and rate are associated with tear breakup time (TBUT), and Ocular Surface Disease Index (OSDI) in dry eye disease [19, 20]. Individuals with dry eyes tend to blink more frequently to improve the tear distribution on the ocular surface [21]. This increased blinking compensates for the reduced tear production and may be caused by discomfort on the ocular surface [22]. In this study, the total blink rate significantly decreased from 8.03 ± 5.04 before epiblepharon surgery to 6.85 ± 4.91 after surgery (*p* = 0.02). The significant decrease in the blink rate after surgery may be related to the alleviation of discomfort from postoperative eyelash irritation. Additionally, this could be interpreted as a reduction in the compensatory mechanism due to a decrease in tear film instability.

Astigmatism is also associated with epiblepharon. There have been conflicting reports regarding changes in astigmatism after surgery, with some studies suggesting a decrease, while others showing no significant difference [2, 23–25]. In our study, corneal astigmatism significantly decreased by 1.23D only in the high astigmatism group, while it significantly increased by 0.34D in the low astigmatism group. The substantial change in corneal astigmatism observed in the high astigmatism group in our study aligns with the findings of Kim et al. [24] who observed a correlation between higher preoperative astigmatism levels and substantial postoperative changes in astigmatism.

There is a hypothesis that epiblepharon can exacerbate astigmatism through tension-inducing mechanisms [24]. In the high astigmatism group, this significant tension might be alleviated by epiblepharon surgery, resulting in a notable improvement in astigmatism. However, in the low astigmatism group, this effect may be minimal.

Additionally, in patients with epiblepharon, initial astigmatism measurements are often unreliable due to corneal erosion. After surgery, as these issues are resolved, more accurate astigmatism measurements can be obtained, likely resulting in changes compared to previous measurements. These results suggest that corneal erosion caused by epiblepharon may impact the accuracy of topography, potentially normalizing evaluations that were previously overestimated or underestimated due to corneal erosion.

There are several limitations in our study. First, the postoperative observational period was short by 2 months. However, we believe that this is enough to assess the structural impact associated with surgery. Second, owing to the pediatric nature of the patients, there were difficulties in obtaining cooperation during examinations, which could have impacted the quality of meibography, leading to the exclusion of some cases. However, we believe that the impact on the results was minimal, as we excluded cases with difficulty in cooperation or low-quality examinations from the analysis. Finally, given the limited sample size, larger studies are needed to generalize these findings.

## Conclusions

This study suggests that lower eyelid epiblepharon surgery in pediatric patients does not significantly alter the structure and function of the MG. Additionally, stabilizing the ocular surface through surgical correction may have beneficial effects on blink rate and astigmatism. When making surgical decisions, correction surgery for lower eyelid epiblepharon can be performed without worrying about adverse effects on the MG.

## Data Availability

All data generated or analyzed during this study are included in this published article. Nevertheless, if needed, the datasets used and/or analyzed during the current study are available from the corresponding authors upon reasonable request.
